# Effects of a Cod Protein Hydrolysate Supplement on Symptoms, Gut Integrity Markers and Fecal Fermentation in Patients with Irritable Bowel Syndrome

**DOI:** 10.3390/nu11071635

**Published:** 2019-07-17

**Authors:** Hanna Fjeldheim Dale, Caroline Jensen, Trygve Hausken, Jan Gunnar Hatlebakk, Ingeborg Brønstad, Jørgen Valeur, Dag Arne Lihaug Hoff, Gülen Arslan Lied

**Affiliations:** 1 Centre for Nutrition, Department of Clinical Medicine, University of Bergen, 5009 Bergen, Norway; 2 Division of Gastroenterology, Department of Medicine, Haukeland University Hospital, 5021 Bergen, Norway; 3 National Centre of Functional Gastrointestinal Disorders, Haukeland University Hospital, 5021 Bergen, Norway; 4 Unger-Vetlesen Institute, Lovisenberg Diaconal Hospital, 0440 Oslo, Norway; 5 Department of Gastroenterology, Oslo University Hospital, 0450 Oslo, Norway; 6 Division of Gastroenterology, Department of Medicine, Ålesund Hospital, Møre & Romsdal Hospital Trust, 6017 Ålesund, Norway; 7 Department of Clinical and Molecular Medicine, Faculty of Medicine and Health Sciences, Norwegian University of Science and Technology, 7491 Trondheim, Norway

**Keywords:** irritable bowel syndrome, bioactive fish peptide, short-chain fatty acids, low-grade inflammation, gut integrity markers

## Abstract

Peptides from fish may beneficially affect several metabolic outcomes, including gut health and inflammation. The effect of fish peptides in subjects with irritable bowel syndrome (IBS) has not previously been investigated, hence this study aimed to evaluate the effect of a cod protein hydrolysate (CPH) supplement on symptom severity, gut integrity markers and fecal fermentation in IBS-patients. A double-blind, randomized parallel-intervention with six weeks of supplementation with 2.5 g CPH (*n* = 13) or placebo (*n* = 15) was conducted. The outcomes were evaluated at baseline and the end of the study. The primary outcomes were symptom severity evaluated by the IBS severity scoring system (IBS-SSS) and quality of life. The secondary outcomes included gut integrity markers and pro-inflammatory cytokines in serum, fecal fermentation measured by concentration of short-chain fatty acids (SCFAs) and fecal calprotectin. The groups were comparable at baseline. The total IBS-SSS-scores were reduced in both the CPH-group (298 ± 69 to 236 ± 106, *p* = 0.081) and the placebo-group (295 ± 107 to 202 ± 103, *p* = 0.005), but the end of study-scores did not differ (*p* = 0.395). The concentrations of serum markers and SCFAs did not change for any of the groups. The baseline measures for the whole group showed that the total SCFA concentrations were inversely correlated with the total IBS-SSS-score (*r* = −0.527, *p* = 0.004). Our study showed that a low dose of CPH taken daily by IBS-patients for six weeks did not affect symptom severity, gut integrity markers or fecal fermentation when compared to the placebo group.

## 1. Introduction

Irritable bowel syndrome (IBS) is a functional gastrointestinal disorder affecting between 10–20% of the population [1], characterized by abdominal pain, bloating and/or distention, constipation and/or diarrhea [2,3]. The pathophysiological mechanisms behind the condition are not fully understood but are suggested to include a combination of genetics, diet, abnormal gut microbiota, abnormal gut endocrine cells, increased intestinal permeability and low-grade inflammation [4,5].

Diet is considered as an important factor in IBS, with over half of the patients reporting worsening of symptoms in relation to intake of certain foods [6]. The effects of different sources of carbohydrates have been investigated in patients with IBS. Further, a diet low in fermentable oligo-, di-, monosaccharides and polyols (FODMAPs) is currently one recommended dietary treatment [2]. FODMAPs are poorly digested in the small intestine. When reaching the colon fermentation of these carbohydrates by colonic bacteria, this leads to the generation of gases and short chain fatty acids (SCFAs). The levels of these metabolic end-products are altered in patients with IBS, conceivably causing symptoms [7,8]. The SCFAs exert several important physiological functions, such as influencing pathways involved in the gene regulation of the metabolism, inflammation and disease, as well as protect against diseases in the gastrointestinal tract [9]. A diet low in FODMAPs is associated with distinct alterations in the composition and function of the gut microbiota, and hence the levels of SCFAs. The long-term effect of the diet has not been well established [10,11].

Some dietary proteins are a source of bioactive peptides, exerting specific effects extending beyond the mere nutrient supply. These peptides can occur naturally by digestion in the gut or be consumed as already hydrolyzed proteins in dietary supplements [12]. Several animal studies have suggested that bioactive peptides from hydrolyzed fish proteins may beneficially influence health by improving the lipid profile, body composition and glucose metabolism [13,14,15,16]. This is supported by increasing evidence from recent clinical trials in human subjects, suggesting that supplements containing fish protein hydrolysates may beneficially influence several metabolic outcomes [17,18,19,20,21]. In addition, it is suggested that fish protein hydrolysates may have an immune-modulating effect with beneficial properties in the gut [22,23]. A chronic low-grade mucosal inflammation and increased intestinal permeability have been assumed to contribute to symptom generation in IBS patients, and several gut integrity markers have been investigated as potential biomarkers. These include zonulin, a physiologic regulator of intercellular tight junctions and suggested as a marker for impaired gut-barrier function [24]; lipopolysaccharide-binding protein (LBP), an acute phase protein suggested as a marker of bacterial translocation [25] and an intestinal fatty acid binding protein (iFABP), a marker for intestinal epithelial cell damage [26]. The clinical implications of these gut integrity markers in IBS have not been established. However based on the hypothesis of fish protein hydrolysate as a possible modulator of the gut, they can be relevant for evaluation in combination with pro-inflammatory cytokines.

The evidence and knowledge are limited on the specific impact of different sources of proteins in patients with IBS. According to clinical experience, IBS symptoms are most often linked to the digestion of carbohydrates, and further, dietary proteins are normally well tolerated. Investigations of different dietary sources of protein in healthy individuals have indicated that they affect the diversity and composition of the human gut microbiota in different degrees [27]. Recent results indicate that the presence of fish proteins in the diet have an impact on the composition and activity of the gut microbiome, influencing the microbiota composition [28]. To the authors’ knowledge, the effect of a peptide supplement in IBS patients has previously not been reported. The environmental and economic benefits of expanding the utilization of by-products from the fishing industry, in addition to the need for novel, additional dietary treatment strategies for patients with IBS, make this study warranted.

The aim of this study was to evaluate the effect of a supplement with cod protein hydrolysate (CPH) on inflammation and gastrointestinal health, including changes in IBS symptoms. For this purpose, this study assessed symptom severity and analyzed gut integrity markers, including pro-inflammatory cytokines in serum, fecal fermentation products (SCFAs) and fecal calprotectin in patients with IBS.

## 2. Materials and Methods

### 2.1. Patients

The patients were recruited through advertisements on the internet between December 2018 and January 2019. The potential subjects answering the online recruitment form were interviewed for general eligibility and compliance with inclusion and exclusion criteria by telephone. The suitable candidates then received more information and signed the informed consent form. A 3-day dietary record and equipment to collect a baseline stool sample was sent by post to the participants prior to the baseline visit at the hospital.

The inclusion criteria were age 20–70 years, BMI 18–30 kg/m^2^ and IBS diagnosis according to Rome IV criteria with predominant diarrhea (IBS-D) or mixed bowel movements (IBS-M). The exclusion criteria were fish allergy, diabetes mellitus, elevated blood pressure, chronic diseases (that might affect the evaluation of the study outcomes), acute infections, substance abuse, immunocompromised patients defined as taking immuno-suppressive medications, patients eating a strict low-FODMAP diet, use of antibiotics during the last 4 weeks before the inclusion or use of medications for the IBS diagnosis.

### 2.2. Study Design and Protocol

The study was a double-blinded, randomized parallel group trial, and included a six-week intervention with a drink containing 2.5 g CPH (test material) or 2.5 g maltodextrin (placebo). CPH or placebo powder was delivered to the patients in sealed bags containing doses for one day. The patients mixed the powder with water and drank it at least 10 min before breakfast each morning.

The baseline visit included assessment of medical data, IBS-diagnosis and biochemical variables. The Rome IV criteria were used to confirm the clinical diagnosis of IBS [29]. The study outcomes were evaluated at baseline and at the end of the study. The primary outcomes were symptom severity evaluated by IBS severity scoring system (IBS-SSS) and quality of life (QoL). The secondary outcomes included gut integrity markers and pro-inflammatory cytokines in serum, fecal fermentation measured by a concentration of short-chain fatty acids (SCFAs) and fecal calprotectin. All subjects completed a dietary record for three days prior to taking the fecal sample at baseline and at the end of the study. The subjects were instructed not to make any changes in the diet while attending the study, and not to take any nutritional supplements containing omega-3 or pre- or probiotics for 6 weeks before the study start, and during the study.

The study was conducted according to the guidelines laid down in the Declaration of Helsinki and the Regional Committees for Medical and Health Research Ethics of Central Norway (2018/1825) approved all procedures involving human. All subjects gave written informed consent and the trial was registered at clinicaltrials.gov as NCT03801057.

### 2.3. Test Material

The test material was a lemon-flavored powder provided from the manufacturer (Firmenich Bjørge Biomarin AS, Ålesund, Norway) in standardized sealed plastic-coated aluminum bags containing 8 g powder to be mixed with 100 mL cold water. Each powder bag contained 5 g glucose monohydrate, 0.0025 g Tastegram Powder Flavor, 0.1 g Lemongrass Durarome taste and 0.7 g citric acid, in addition to 2.5 g of CPH or maltodextrin (placebo). The thorough tests assured that it was not possible to identify the active ingredient from placebo, according to the flavor or the appearance.

The cod protein hydrolysate powder was made by Firmenich Bjørge Biomarin AS by hydrolyzing fish meat of Atlantic cod (*Gadus morhua*) with Protamex^®^ (Novozymes AS) followed by spray drying of the soluble part of the enzyme digest. The CPH raw material contained approximately 89% protein by weight, <0.2% fat, 0% carbohydrate, <3.0% water, 10% ash, 0.1% NaCl, 1.7% sodium and 0.07% chloride. The free amino acids accounted for 4.77% of the total amino acids in the cod protein hydrolysate (CPH), and the ratio essential amino acids/non-essential amino acids was 0.70. The analysis of the molecular weight distribution showed that approximately 90% of the peptides in the CPH had a molecular weight of 2000 Daltons (Da) or less (18 amino acids or less), approximately 75% of 1000 Da or less (10 amino acids or less), while approximately 55% had a molecular weight of 500 Da or less (5 amino acids or less). Approximately 25 to 30% of the peptides had a molecular weight less than 200 Da, which represents small dipeptides and free amino acids. The production process and composition of the CPH raw material has been described in detail in a previous publication [17].

The patients were randomly assigned to the experimental (CPH) or the control (placebo) group. Randomization was completed using a computer-based automated sequence implemented in the digital central case-report file (webCRF). The randomization sequence was generated by a person blinded to the assignment of patients to the study groups. The random assignment order was created using block randomization. The powder bag was coded by a person blinded to the allocation of patients. Both patients and study investigators were unaware of the study-group allocation (double-blinded study). The key of randomization was provided to the investigators when the trial had ended, and the statistical analysis was completed.

### 2.4. Blood Samples

The blood samples were taken at baseline and after the six-week intervention. General biochemical tests were taken for safety purposes (albumin, prealbumin, vitamin-B12, vitamin-D, leucocytes, thrombocytes, hemoglobin, HbA1_c_, CRP, sodium, potassium, ALAT, ALP, creatinine and ASAT). The samples were analyzed according to standard accredited methods at the Laboratory for Clinical Biochemistry, Haukeland University Hospital and Department of Medical Biochemistry, Ålesund Hospital.

The gut integrity markers measured in the serum included iFABP, LBP and zonulin. In addition, this study analyzed the following pro-inflammatory cytokines in serum—tumor necrosis factor alpha (TNF-α), interferon gamma (INF-γ) and interleukins (IL-4, 6, 8, 10). The serum was obtained by centrifugation of full blood at 2000× *g* in room temperature (20 °C) for 10 min after 30–60 min of coagulation, using serum separator cloth activator tubes. The samples were aliquoted and stored at −80 °C until analysis. The analyses of cytokines were performed by a cytokine human ultrasensitive magnetic 10-plex panel for Luminex^TM^ platform, Cat# LHC6004 (Invitrogen, Thermo Fisher Scientific, Waltham, MA, USA). Furthermore, iFABP was analyzed by Human FABP2 (intestinal) ELISA kit Cat# EHFABP2 (Invitrogen, Thermo Scientific, Waltham, MA, USA), LBP was analyzed by Human LBP (Lipopolysaccharide-binding protein) ELISA kit, Cat# EKH3120 (Biosite, Taby, Sweeden) and zonulin was analyzed by IDK^®^ Zonulin ELISA, Ref# K5601 (Immun diagnostic, Bensheim, Germany).

### 2.5. Fecal Samples

The fecal samples for analyses of calprotectin and SCFAs were collected before and after the six-week intervention. The patients were instructed to freeze the samples immediately after collection at home (−20 °C freezer) and bring the samples frozen to the hospital visits. The samples were stored at −20 °C until analysis.

The fecal samples for evaluation of calprotectin were collected in Calpro Easy Extract containers (Calpro AS, Oslo, Norway) and calprotectin content was measured using CALPROLAB^TM^ Calprotectin ELISA (ALP) CALP0170 (CALPROLAB, Calpro AS, Oslo, Norway).

The fecal samples for evaluation of SCFAs were collected in designated containers (Sarstedt AG & Co., product No. 80.734.301, Numbrecht, Germany). Upon analysis, 0.5 g of the fecal material was added to distilled water containing 3 mmol/L of 2-ethylbutyric acid (as internal standard) and 0.5 mmol/L of H_2_SO_4_. 2.5 mL and then homogenized. After homogenization, 2.5 mL of the sample was vacuum distilled, according to the method of Zijlstra et al. [30], as modified by Hoverstad et al. [31]. The distillate was analyzed with gas chromatography (Agilent 7890 A, Calif., USA), using a capillary column (serial No. USE400345H, Agilent J&W GC Columns, Calif., USA) and quantified using internal standardization. Flame ionization detection was employed. The fecal samples were analyzed for both major SCFAs (acetic, propionic and butyric) and minor SCFAs (iso-butyric, valeric, iso-valeric, capronic and iso-capronic acids). The results were expressed in mmol/kg wet weight.

### 2.6. Symptom Questionnaires

The symptoms related to the IBS diagnosis were assessed by symptom questionnaires at baseline and after the six-week intervention. The severity of abdominal symptoms was assessed by the validated IBS severity scoring system (IBS-SSS). The maximum score is 500 points, with the following grading: Mild (75–175 points), moderate (175–300) and severe (>300 points). A reduction of 50 points or more in the IBS-SSS questionnaire is regarded as clinically relevant [32]. The Quality of life (QoL) was evaluated by the validated Short Form-Nepean Dyspepsia Index (SF-NDI) with a maximum sum score of 50 points [33].

### 2.7. Estimation of Nutritional Intake

The calculations of energy and macronutrient intake, as well as FODMAP content in the diet, were performed using the Nordic nutrient calculation program Dietist Net Pro (Bromma, Sweden). The estimations reported were the mean daily intake based on three days of dietary records registered at baseline and during the last days of the six-week intervention. The total FODMAP content is the sum of calculated fructose, fructose in excess of glucose, lactose, fructans, polyols, fructo-oligosaccharides (FOS) and galacto-oligosaccharides (GOS).

### 2.8. Statistical Analysis

The statistical analyses were conducted in SPSS data package (SPSS Statistics 24.0, IBM Company, Armonk, NY, USA) and GraphPad Prism version 7.0. (GraphPad Software, Inc., San Diego, CA, USA). For all graphical work, this study used GraphPad Prism. The data are presented as the mean ± SD, unless otherwise stated. To compare the differences between the baseline and the end of the study measures for each subject, paired *t*-tests, and unpaired *t*-tests were used comparing differences between the CPH and the placebo group. The assessment of correlations was completed with Pearson’s correlation coefficient. All tests were two-sided and *p*-values < 0.05 were considered statistically significant.

A power calculation for estimation of the sample size was not performed. According to protocol, this study intended to include 30 patients.

## 3. Results

### 3.1. Patient Characteristics

**Figure 1:**
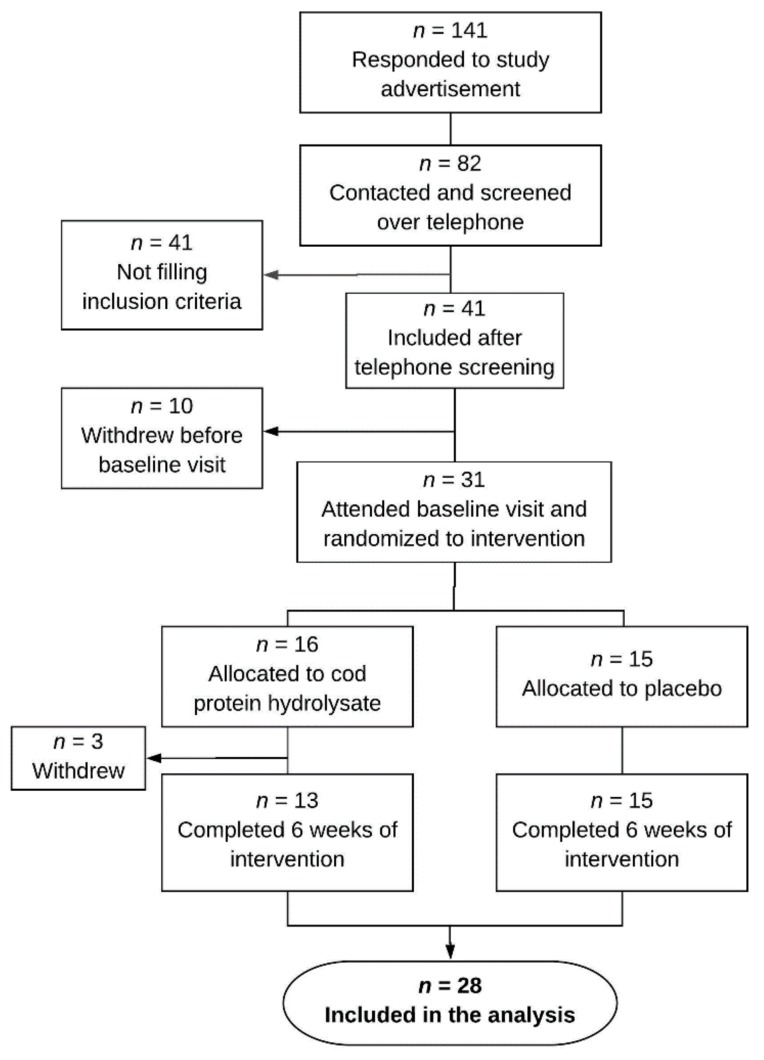
Flow-chart describing the inclusion process of the 28 irritable bowel syndrome (IBS) patients completing the six-week trial and included in the analysis.

Thirty-one eligible patients were included, of whom 28 patients (23 women and 5 men) completed the trial and were included in the analyses. Three patients withdrew after randomization, one patient due to disliking the supplement and two patients due to experiencing increased diarrhea. The inclusion process is showed in Figure 1. According to the Rome IV phenotype definition, 19 patients were classified as diarrhea-predominant (IBS-D) and 9 patients as mixed bowel habits (IBS-M). Ten patients reported to avoid specific high-FODMAP food items they experienced as problematic (e.g., lactose, apples, wheat and/or garlic). In accordance with the inclusion criteria, no subjects followed a strict low FODMAP diet. According to total IBS-SSS scores at baseline, IBS severity was classified as mild in 2 patients, moderate in 9 patients and severe in 17 patients. The groups were comparable at baseline, except for a significant difference in BMI. Table 1 elucidates the baseline characteristics.

### 3.2. Irritable Bowel Syndrome Symptom Scores and Quality of Life

**Figure 2:**
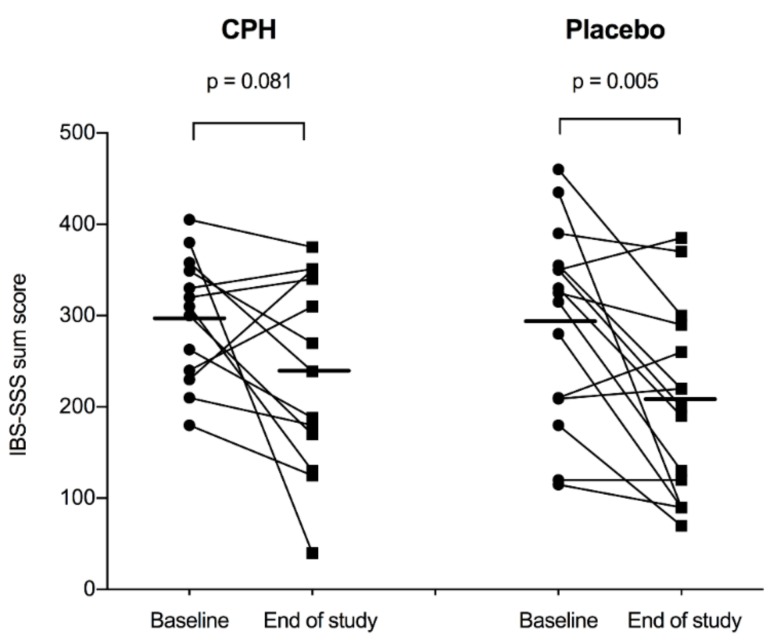
IBS-SSS scores at baseline and after the six-week intervention for the cod protein hydrolysate (CPH) group (*n* = 13) and the placebo-group (*n* = 15). The horizontal lines show the mean values.

**Figure 3:**
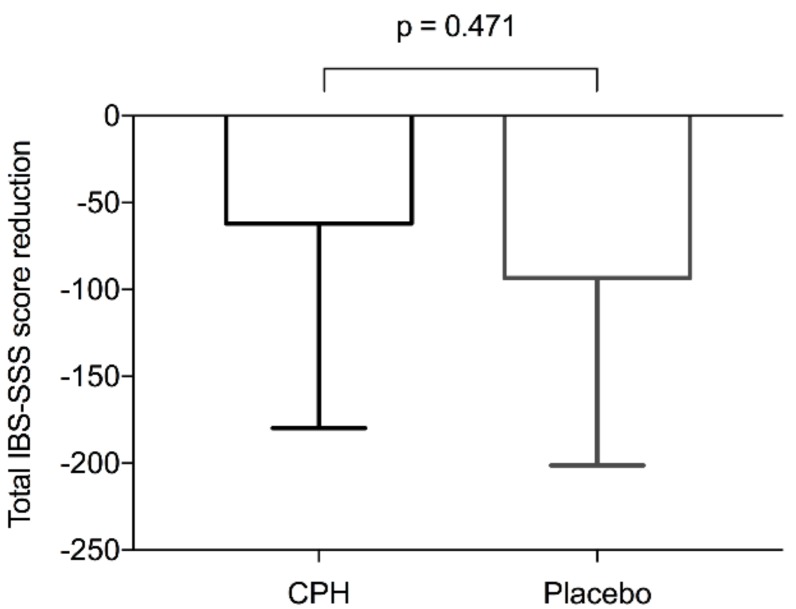
The reduction in total IBS-SSS scores from baseline to after the six-week intervention for the cod protein hydrolysate (CPH) group (*n* = 13) and the placebo-group (*n* = 15) expressed as the mean difference from baseline.

Table 2 reports symptom scores. According to total IBS-SSS scores, IBS symptoms improved from baseline to after six weeks of intervention in both the CPH-group (from 298 ± 69 to 236 ± 106, *p* = 0.081) and the placebo-group (from 295 ± 107 to 202 ± 103, *p* = 0.005) (Figure 2). Regarding the mean difference from baseline to after the intervention, the total IBS-SSS score did not differ significantly between the CPH-group (−62 ± 118) and the placebo-group (−93 ± 108, *p* = 0.471) (Figure 3). After the intervention, the scores did not differ significantly between the groups (*p* = 0.395).

All IBS-SSS sub scores (pain severity, pain frequency, bloating, bowel habit dissatisfaction and life interference) declined from baseline to the end of the study in both groups. For the placebo-group, all symptoms declined significantly, whereas for the CPH-group, only bloating and life interference significantly declined. Significant differences between the groups for any other of the reported symptoms, either at baseline or at the end of the study, did not occur. The baseline measures for the whole group (*n* = 28) showed no significant correlations between total IBS-SSS scores and the calculated total FODMAP content in the diet.

The scores for QoL declined in both groups from baseline to the end of the study, with a significant reduction in the placebo-group (Table 2). The scores did not differ between the groups either at baseline (*p* = 0.191) or after the intervention (*p* = 0.094).

### 3.3. Gut Integrity Markers and Pro-Inflammatory Cytokines in Serum

The values for gut integrity markers and pro-inflammatory cytokines in serum are shown in Table 3. No significant changes in concentrations of LBP, iFABP or zonulin (ng/mL) were observed between baseline and the end of the study, in either the CPH-group or the placebo-group. The levels of zonulin were significantly lower for the CPH-group than the placebo-group at baseline (*p* = 0.011), but no other differences were observed between the groups. For the analyzed pro-inflammatory cytokines, only IL-8 showed values within detectable range, thus no data are reported for IL-4, 6 10, TNF-α and INF-γ. The concentration of IL-8 (pg/mL) increased from baseline to the end of the study in both groups, but the increase was not significant, and no differences were observed between the groups.

The baseline measures for the whole group (*n* = 28) showed no significant correlations between the serum markers and the total IBS-SSS score.

### 3.4. Fecal SCFAs

**Figure 4:**
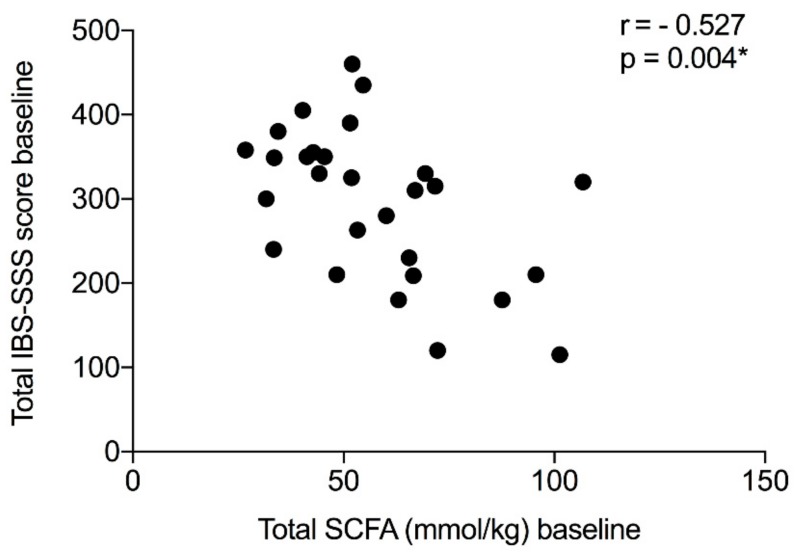
The relationship between the total concentration of SCFAs and the total IBS-SSS score at baseline for 28 patients with IBS. * The fecal total SCFA concentrations at baseline were statistically significant inversely correlated with the IBS-SSS baseline sum score.

This study observed no significant changes in concentrations of any SCFAs between the baseline and the end of the study measures, either in the CPH-group or the placebo-group. Table 4 outlines the values. No significant differences were observed between the groups for any of the measured SCFAs, either at baseline or the end of the study. The fecal total SCFA concentrations at baseline for the whole study population were inversely correlated with the IBS-SSS baseline sum score (r = −0.527, *p* = 0.004) (Figure 4). No correlations were observed between the total SCFA concentration and the serum markers or the total FODMAP content in the diet for the whole group at baseline.

### 3.5. Fecal Calprotectin

No significant changes in concentration of fecal calprotectin was observed between the baseline and the end of the study measures, either in the CPH-group (baseline: 137 ± 213 mg/kg, after intervention: 129 ± 134 mg/kg, *p* = 0.216) or the placebo-group (baseline: 117 ± 248, end of study: 99 ± 157, *p* = 0.525). Numerically, the mean value decreased slightly from baseline to the end of the study for both groups. No significant differences were observed between the groups either at baseline (*p* = 0.525) or at end of the study (*p* = 0.496).

### 3.6. Dietary Records

The comparison between the mean daily nutrient intake (kcal, proteins (g/kg body weight), carbohydrates (g), fiber (g), total FODMAPs (g), fat (g) and alcohol (g)) revealed no differences between the two groups either at baseline or at end of the study. No significant changes in nutrient intake from baseline to the end of the study were observed within each group.

### 3.7. Adverse Events

Three subjects allocated to CPH supplement withdrew after inclusion. One did not like the smell of the supplement, thus reported nausea associated to consumption. Two subjects reported an increase in IBS symptoms related to diarrhea and/or pain, of which one related the increase in symptoms to the supplement, whereas one acknowledged the symptoms as a regular bad IBS period.

## 4. Discussion

To the authors’ knowledge, this is the first study reporting on the effects of a fish peptide supplement in IBS patients. The study was designed to investigate the effect of a dietary supplement with cod protein hydrolysate, hypothesized to contain bioactive peptides with potentially beneficial properties in the gut. This study observed no significant effects of the supplement for any of the outcome measures, when compared to the placebo.

The lack of effects could be explained by several factors. It was a reduction in symptom scores (primary outcome) during intervention in both the CPH and the placebo group, with a significant reduction in the total score only in the placebo group, and no significant differences between groups. Interventions in IBS patients are likely to be influenced by a strong placebo or nocebo effect [34]. As this study did not observe any changes in secondary outcomes to support an effect of the CPH supplement, it was assumed that the symptom reduction in both groups can be attributed a placebo effect, caused by the patients’ expectations on symptom improvement when taking a dietary supplement in a clinical trial. It is possible that a different effect may have been observed if the hydrolysate was given in a higher dose. Previous studies investigating the health effects from a supplement containing hydrolyzed proteins from fish in human individuals have reported beneficial metabolic effects in a low dose range of 1 to 6 g a day [17,18,19,20]. Based on this, the authors chose an intervention with 2.5 g per day. This dose is negligible per se when put in context with the total daily dietary protein intake. Thus, if an effect were to be observed, our hypothesis was that it could have been attributed to the CPH.

According to our findings, no changes were observed in SCFA concentration after the intervention, but there was an inverse correlation between the total SCFA concentration at the baseline and the total IBS-SSS score when looking at the whole study population. This indicated that those with higher concentrations of SCFAs have less IBS-related symptoms. Previous studies investigating alterations in SCFA concentrations in IBS patients have reported inconsistent results. However, differences in fecal SCFA concentrations have been reported between IBS patients and healthy controls [35]. In addition, altered concentrations of both SCFAs and cytokines have been observed in response to a low-FODMAP diet in IBS patients [36,37]. The clinical relevance of a change in fecal SCFA concentration is currently not known. The fecal fermentation is dependent on both diet and gut microbiota, and the primary source for colonic production of SCFAs is low-digestible carbohydrates [37]. As the intake of carbohydrates did not change during the intervention, the lack of distinct findings in the current trial was not surprising.

The authors hypothesized that the CPH might influence inflammation and gut permeability, hence pro-inflammatory cytokines and gut integrity markers were evaluated. A change in either gut integrity markers or pro-inflammatory cytokines in response to intervention was not observed. Based on the theory of increased gut permeability and low-grade inflammation as a central contributor to IBS etiology, several studies have compared a broad range of gut integrity markers and inflammatory markers in IBS patients to healthy controls, aiming to identify possible biomarkers. To date, the findings have been inconsistent, but a reported tendency has been altered levels of gut integrity markers [24,26,38] and higher levels of pro-inflammatory cytokines [39,40,41] in IBS patients compared to healthy controls. Interestingly, of the analyzed cytokines, only IL-8 showed values within a detectable range at baseline, suggesting that neither IL-4, IL-6, IL-10, TNF-α or INF-γ are relevant as inflammatory markers for our IBS population with predominant diarrhea or mixed bowel habits. However, the measured levels of fecal calprotectin, with the mean baseline levels of above 100 mg/kg and levels above 50 mg/kg regarded as a positive value, support the assumption of low-grade inflammation as a contributor to disease. Hence, other inflammatory markers other than the cytokines investigated in this trial might be of interest in future studies.

The potential beneficial effects of dietary supplements with peptides and amino acids are in general not well investigated in IBS populations. Interestingly, Zhou et al. recently reported that dietary supplementation with the essential amino acid glutamine significantly improved symptoms in patients with post-infectious, diarrhea-predominant IBS [42]. Supplementation with glutamine (5 g per day for eight weeks) was found to restore the intestinal permeability, leading to a reduction of diarrhea and abdominal pain, compared to the control (whey protein). Notably, the CPH used in our study holds a low concentration of glutamine, 0.78 mg/g CPH, corresponding to 1.95 mg glutamine per day with a dose of 2.5 g CPH (data on composition of the CPH are reported in a previous publication [17]).

The design holds some limitations. The cohort of the patients studied included only IBS-D and IBS-M subtypes. The absence of the effect of the intervention could be due to either the small cohort of the patients studied or to the low dose used. A larger cohort, including all the IBS subtypes, is needed before drawing any conclusion.

## 5. Conclusions

In summary, no effects of a supplement with 2.5 g CPH given daily for six-weeks was observed on symptom severity, gut integrity markers, pro-inflammatory cytokines or fecal fermentation products in a small group of patients with IBS, when compared to the placebo. Future studies should aim to target low-grade inflammation and evaluate the potential effect of supplementation with peptides containing bioactive sequences with known anti-inflammatory properties in IBS patients.

## Figures and Tables

**Table 1:** The baseline characteristics of 28 irritable bowel syndrome (IBS) patients randomly allocated to six weeks supplementation with either cod protein hydrolysate (CPH) or placebo.

Characteristics	CPH (*n* = 13)	Placebo (*n* = 15)	*p*-Value
Age, years	42.7 (11.9)	45.1 (14.8)	0.647
Gender (male/female)	1/12	4/11	-
BMI, kg/m^2^	24.1 (2.8)	27.2 (3.9)	0.025 *
IBS-D/IBS-M	8/5	11/4	-
IBS severity ^1^ mild	0	2	-
moderate	5	4	-
severe	8	9	-
IBS-SSS sum score (0–500)	295 (107)	298 (69)	0.928
Energy intake, kcal/day	1750 (500)	1950 (395)	0.245
Protein intake, g/kg BW/day	1.2 (0.4)	1.0 (0.3)	0.185
Carbohydrates, g/day	140.0 (68.1)	180.2 (53.6)	0.093
Fiber, g/day	18.9 (7.6)	17.9 (5.7)	0.697
Total FODMAP ^2^, g/day	11.2 (6.6)	13.0 (11.3)	0.623
Alcohol, g/day	5.3 (6.8)	6.2 (8.6)	0.760
Fat, g/day	130.0 (190.5)	91.7 (22.6)	0.445

BW: body weight; BMI, Body Mass Index; IBS-D, IBS with diarrhea; IBS-M, mixed IBS; FODMAP, fermentable oligo-, di-, monosaccharides and polyol; IBS-SSS, IBS severity scoring system. ^1^ IBS severity based on the baseline IBS-SSS sum score: mild (75–175 points), moderate (175–300) and severe (>300 points); ^2^ Total FODMAP content in the diet based on mean daily intake from 3-days dietary records. * Statistically significant difference between the groups.

**Table 2:** Symptoms scores at baseline and after the six-week intervention for IBS patients in the cod protein hydrolysate (CPH) group and the placebo-group.

		CPH (*n* = 13)			Placebo (*n* = 15)		
Symptom Scores		Baseline	End of Study	*p*-Value	Baseline	End of Study	*p*-Value
IBS-SSS	Sum score	298.1 (68.9)	236.0 (105.9)	0.081	294.9 (106.6)	201.7 (103.6)	0.005 *
	Pain severity	45.0 (25.1)	39.2 (25.3)	0.096	43.3 (33.3)	25.0 (32.3)	0.016 *
	Pain frequency	45.4 (34.3)	39.2 (25.3)	0.446	47.3 (35.5)	25.3 (28.5)	0.018 *
	Bloating	65.9 (18.5)	46.0 (27.5)	0.046 *	59.3 (35.9)	37.0 (31.1)	0.038 *
	Bowel habit dissatisfaction	77.1 (20.1)	62.3 (30.8)	0.059	73.7 (26.2)	54.0 (30.1)	0.034 *
	Life interference	78.5 (17.1)	57.4 (30.4)	0.023 *	71.3 (23.6)	60.3 (22.2)	0.034 *
SF-NDI	Sum score	28.0 (7.1)	23.9 (9.1)	0.104	24.1 (7.9)	18.3 (7.9)	0.042 *

IBS-SSS: Irritable bowel syndrome severity scoring system, SF-NDI: Short Form-Nepean Dyspepsia Index, * Statistically significant difference between the baseline score the and end of study score.

**Table 3:** The concentrations of gut integrity markers and pro-inflammatory cytokines in serum samples collected before and after six weeks of supplementation with cod protein hydrolysate (CPH) or placebo.

	CPH (*n* = 13)			Placebo (*n* = 15)		
Inflammatory Marker	Baseline	End of Study	*p*-Value	Baseline	End of Study	*p*-Value
iFABP (ng/mL)	68.3 (43.2)	58.2 (28.0)	0.432	55.5 (20.1)	56.2 (28.7)	0.940
LBP (ng/mL)	6097 (2630)	6446 (2043)	0.355	6931 (3023)	6884 (3274)	0.925
Zonulin (ng/mL)	40.5 (5.6)	42.5 (6.3)	0.125	46.6 (5.9)	45.7 (5.3)	0.286
IL-8 (pg/mL)	8.8 (11.8)	11.4 (10.1)	0.185	7.4 (6.5)	8.9 (9.1)	0.413

iFABP: Intestinal fatty acid binding protein, LBP: Lipopolysaccharide binding protein, IL: Interleukin.

**Table 4:** Short-chain fatty acid (SCFA) concentrations (mmol/kg) in fecal samples collected before and after six weeks of supplementation with cod protein hydrolysate (CPH) or the placebo.

	CPH (*n* = 13)			Placebo (*n* = 15)		
Parameter	Baseline	End of Study	*p*-Value	Baseline	End of Study	*p*-Value
Total SCFA	51.8 (22.4)	55.7 (24.1)	0.591	62.6 (19.5)	62.4 (23.1)	0.997
Acetic acid	30.4 (12.3)	32.2 (14.5)	0.705	36.3 (11.9)	35.9 (11.3)	0.921
Propionic acid	9.9 (6.3)	10.4 (6.7)	0.768	10.7 (3.8)	10.8 (6.0)	0.963
Butyric acid	7.4 (3.9)	8.5 (5.0)	0.473	10.3 (4.5)	10.0 (5.1)	0.827
Iso-butyric acid	1.1 (0.5)	1.3 (0.6)	0.257	1.3 (0.7)	1.4 (0.8)	0.595
Valeric acid	1.1 (0.8)	1.2 (0.6)	0.785	1.7 (0.9)	1.8 (1.0)	0.805
Iso-valeric acid	1.6 (0.8)	1.9 (1.0)	0.322	1.9 (1.1)	2.0 (1.3)	0.554
Caproic acid	0.3 (0.5)	0.3 (0.4)	0.992	0.5 (0.5)	0.6 (0.8)	0.425
Iso-caproic acid	0.0 (0,0)	0.0 (0.0)	-	0.01 (0.04)	0.01 (0.04)	0.670
